# *Journal of Medical Radiation Sciences* – a joint journal between Australia and New Zealand

**DOI:** 10.1002/jmrs.7

**Published:** 2013-02-03

**Authors:** Cherry Agustin

**Affiliations:** Journal of Medical Radiation Sciences

## Introduction

**Figure 1:**
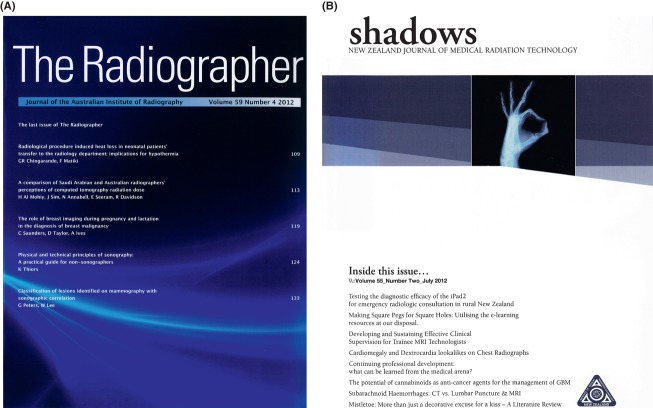
(A) 2012 cover page of *The Radiographer* and (B) 2012 cover page of *Shadows*.

Welcome to the first issue of *Journal of Medical Radiation Sciences* (JMRS). The JMRS is a joint journal publication of the Australian Institute of Radiography (AIR) and New Zealand Institute of Medical Radiation Technology (NZIMRT). The JMRS constitutes the amalgamation of *The Radiographer* in Australia and *Shadows* in New Zealand (Fig. 1A and B).

### The Radiographer and Shadows

**Figure 2:**
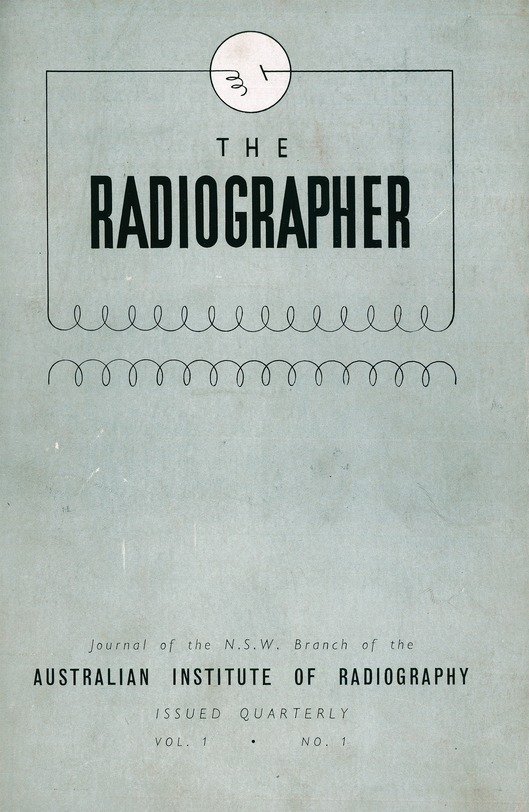
Australia: 1948 cover page of *The Radiographer*.

In Australia, *The Radiographer* was first published in 1948 as the Journal of the NSW Branch of the Australian Institute of Radiography (Fig. 2). It later became the Official Journal of the Australasian Institute of Radiography and later changed to the AIR.

**Figure 3:**
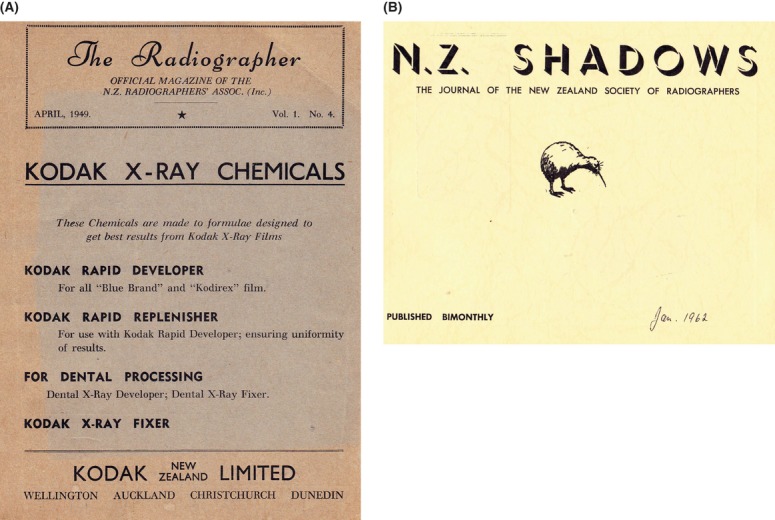
New Zealand: (A) 1949 cover page of *The Radiographer* and (B) 1962 cover page of *Shadows*.

In New Zealand, *Shadows* was previously known as *The Radiographer* between 1948 and 1961. The name was changed to *Shadows* in 1962 (Fig. 3). The journal was initially the publication of the New Zealand Radiographers Association, which later changed over the years to the Journal of the New Zealand Institute of Medical Radiation Technology.

For ease in the transition from having two separate journals to a combined journal, the members of the Editorial Review Board (ERB) of *The Radiographer* and *Shadows* joined to form one ERB of JMRS.

## Content of JMRS

There were many possible journal names suggested by members of the AIR and NZIMRT. The name that received the most votes from members of the ERB was JMRS. This was approved by both boards of the AIR and NZIMRT and officially became the JMRS on 3 September 2012.

*Journal of Medical Radiation Sciences*, as the name implies, encompasses the three strands of diagnostic radiography, radiation therapy, and nuclear medicine. Diagnostic radiography includes medical imaging modalities that utilize x-rays plus sonography and magnetic resonance imaging.

Radiation therapy encompasses all the techniques and technologies in external beam radiation therapy and brachytherapy. Nuclear medicine and molecular imaging involve the use of radiopharmaceuticals, gamma, and hybrid cameras for the diagnosis and treatment of disease.

The topics covered range from technical, education, and patient care–related issues. The journal aim and scope of JMRS was created to reflect clinical practice where collaboration between members of the multi-disciplinary team occurs (Box 1). Therefore, members of the multi-disciplinary team are highly encouraged to also publish in JMRS. The types of papers accepted for publication in JMRS are listed in Box 2.

**Box 1. *Journal of Medical Radiation* Sciences aim and scope**

*Journal of Medical Radiation Sciences* (JMRS) is an international and multi-disciplinary, peer-reviewed journal that accepts manuscripts related to medical imaging/diagnostic radiography, radiation therapy, nuclear medicine, medical ultrasound/sonography, and the complementary disciplines of medical physics, radiology, radiation oncology, nursing, psychology, and sociology. Manuscripts may take the form of original articles, review articles, commentary articles, technical evaluations, case series, and case studies.

*Journal of Medical Radiation Sciences* promotes excellence in international medical radiation science by the publication of contemporary and advanced research that encourages the adoption of the best clinical, scientific, and educational practices in international communities.

*Journal of Medical Radiation Sciences* is the official professional Journal of the Australian Institute of Radiography (AIR) and the New Zealand Institute of Medical Radiation Technology (NZIMRT).

**Box 2. Types of papers submitted in *Journal of Medical Radiation Sciences***

*Original Article*: Describes a research including a systematic review and meta-analysis, and quality improvement.

*Review Article*: This will usually be at the invitation of the editors.

*Commentary*: Discusses relevant aspects of a practice or an issue. Opinions and recommendations are expressed with the support of evidence from the literature.

*Study Protocol*: The aim of publishing study protocols is to inform readers of research projects that are proposed to commence or are ongoing. The study protocol must have full research ethics approval to be considered for publication.

*Technical Evaluation*: An emerging technology or a new technique in medical radiation sciences.

*How to Do It*: An invited educational article relevant to medical radiation sciences professionals. Unsolicited proposals for How to Do It may be submitted; however, in this case, authors should only send an outline of the proposed paper for initial consideration.

*Case Report/Study*: Describes one of the following:

- Previously unreported interventional technique in a recognized disease.
- Previously unreported, relevant imaging observations on recognized disease or lesion.
- Previously unreported clinical condition.
- Previously unreported complication of a radiological procedure.

*Pictorial Review*: The aim of a pictorial review is to provide an up-to-date visual portrayal of a topical issue, having particular educational value.

*Editorial*: An invited commentary on an article published in the same journal issue.

*Letter to the Editor*: Letters to the Editor intended for publication may be submitted on any matters of interest to readers of *Journal of Medical Radiation Sciences*. A letter commenting on an article that has appeared in a previous issue of *Journal of Medical Radiation Sciences* is welcome.

## JMRS cover

**Figure 4:**
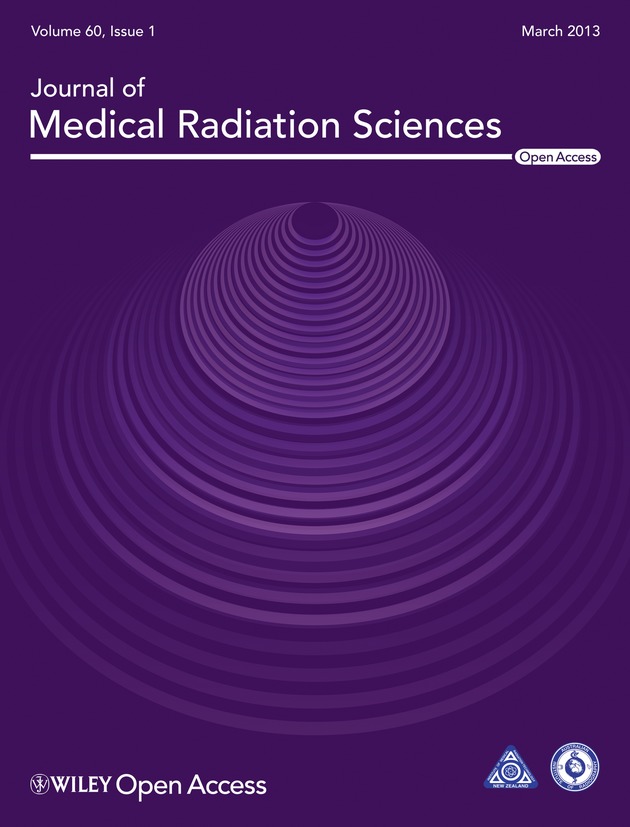
2013 cover page of *Journal of Medical Radiation Sciences*.

Radiation is fundamental to all three strands of medical radiation sciences. The new journal cover (see Fig. 4) depicts the 3D characteristics of radiation, and dark purple was chosen because it was a colour not used by other competitive journals.

## Peer Review and Open Access

The ERB will aim for a median duration of 6 weeks for peer review and for the authors to receive the first decision of their submission. All manuscripts are peer reviewed by two blinded reviewers where possible. The peer reviewers are experts from Australia, New Zealand, and overseas. The decision of the Editor-in-Chief to accept or reject a manuscript for publication is based on the recommendations made by the two expert peer reviewers, the associate editor and deputy editor.

ScholarOne Manuscript platform is a user-friendly manuscript management system. Authors can upload their submissions online and they can monitor the progress of their manuscript at their convenience. The contact person for any question about the journal and the submission process is Angélique Montane, JMRS Editorial Assistant (email: JMRS.EO@wiley.com).

Members of the AIR and NZIMRT will continue to receive printed copies of JMRS. The four issues of JMRS are published in March, June, September, and December. In addition, all approved manuscripts are immediately published online ahead of inclusion in an online or print issue in Wiley-Blackwell's Early View.

*Journal of Medical Radiation Sciences* is an open-access journal, meaning that readers can view or download articles online free of charge. The main advantage of open-access publications is the greater readership compared to those with restricted access or subscription-only journals.^1^

You may have noticed that the first issue of JMRS continues with the volume number of *The Radiographer*. This was done for JMRS to gain entry to PubMed Central as early as possible. Prior to the merge of the two journals, *The Radiographer* was waiting for approval from PubMed Central. It is expected that JMRS will also be indexed to relevant allied health and nursing journal databases.

## Continuing Education of Reviewers and Authors

The members of the ERB and Wiley-Blackwell will provide relevant resources to the reviewers and authors to ensure that the publications are of high quality. The educational resources are listed online on the JMRS website. There will also be publication workshops organized in Australia and New Zealand. We would encourage people to take advantage of this opportunity and participate in a workshop.

## Promote Your Journal

The ultimate success of your journal begins with you, the reader of this journal. You play an important role in promoting your journal to your colleagues in the workplace and especially to the members of the multi-disciplinary team. Inform your university and workplace librarian to include JMRS to their collection. Invite your colleagues in Australia, New Zealand, and overseas to publish in JMRS.

An international advisory panel was appointed to increase the number of contributions from Asia, North America, and Europe. With the implementation of an international advisory panel and with your help in increasing the number of submissions from Australia and New Zealand, we can create a journal that demonstrates excellence in publication.

## The Future

The ERB has many expectations for the future: an increasing number of submissions, an increasing number of local and global readers, an increasing number of citations, an increasing visibility leading to inclusion in Medline, and for JMRS to gain an impact factor. The ERB and the team from Wiley-Blackwell will continue to develop strategic plans to further improve the status of your journal. Readers of JMRS are highly encouraged to provide feedback to further improve our journal by writing a letter to the editor or by contacting the editorial office at JMRS.EO@wiley.com.
